# Transient antiretroviral therapy selecting for common HIV-1 mutations substantially accelerates the appearance of rare mutations

**DOI:** 10.1186/1742-4682-5-25

**Published:** 2008-11-14

**Authors:** Tinevimbo Shiri, Alex Welte

**Affiliations:** 1 School of Computational and Applied Mathematics, University of the Witwatersrand, Private Bag 3, Johannesburg, South Africa; 2 South African Centre of Excellence in Epidemiological Modelling and Analysis (SACEMA), Stellenbosch University, South Africa

## Abstract

**Background:**

Highly selective antiretroviral (ARV) regimens such as single dose nevirapine (NVP) used for prevention of mother to child transmission (PMTCT) in resource-limited settings produce transient increases in otherwise marginal subpopulations of cells infected by mutant genomes. The longer term implications for accumulation of further resistance mutations are not fully understood.

**Methods:**

We develop a new strain-differentiated hybrid deterministic-stochastic population dynamic type model of healthy and infected cells. We explore how the transient increase in a population of cells transcribed with a common mutation (modelled deterministically), which occurs in response to a short course of monotherapy, has an impact on the risk of appearance of rarer, higher-order, therapy-defeating mutations (modelled stochastically).

**Results:**

Scenarios with a transient of a magnitude and duration such as is known to occur under NVP monotherapy exhibit significantly accelerated viral evolution compared to no-treatment scenarios. We identify a possibly important new biological timescale; namely, the duration of persistence, after a seminal mutation, of a sub-population of cells bearing the new mutant gene, and we show how increased persistence leads to an increased probability that a rare mutant will be present at the moment at which a new treatment regimen is initiated.

**Conclusion:**

Even transient increases in subpopulations of common mutants are associated with accelerated appearance of further rarer mutations. Experimental data on the persistence of small subpopulations of rare mutants, in unfavourable environments, should be sought, as this affects the risk of subverting later regimens.

## Background

The rapidity of human immunodeficiency virus (HIV) replication, combined with its high reverse transcriptase error rate [1], leads to rapid viral evolution, in particular the emergence of drug resistance. Treatment that is unable to sufficiently inhibit viral replication allows the appearance and/or selection of drug-resistant strains. Further accumulation of resistant variants may limit therapeutic efficacy and jeorpadize subsequent treatment options.

A single dose nevirapine (NVP) regimen for prevention of mother to child transmission (PMTCT) is a well known example of a suboptimal regimen that inevitably, if temporarily, exerts selective pressure in favour of resistant strains. This is still a major concern in developing countries where a prophylactic regimen of single dose NVP is widely used for PMTCT [2]. Given the high frequency of mutation, some minority resistant mutants are always preexisting, albeit in trace quantities, at the moment therapy is initiated. Because of the long half-life of single dose NVP, with blood levels detectable up to 2–3 weeks after exposure [3,4], the duration of sub-therapeutic NVP concentrations may present a significant hazard of developing resistance for the mother. There is a risk of treatment failure after single dose NVP exposure, if the treatment includes a NNRTI [5]. The question arises whether, and to what extent, a transient treatment-induced boost to an otherwise marginal subpopulation results in increased risk of accumulation of further resistance mutations that could potentially increase the risk of subsequent NNRTI-based treatment failure.

In the search for better PMTCT regimens, improved efficacy has been demonstrated for a number of short course regimens for PMTCT in resource-limited settings. For example, 1) use of single dose NVP with additional short course of zidovudine/lamivudine during 3–7 days postpartum [6], 2) addition of single dose NVP to zidovudine short course during the antenatal period [7] and, recently, 3) use of intrapartum single dose of combined tenofovir/emtricitabine taken after antenatal short course of zidovudine plus intrapartum single dose NVP [8]. These regimens improve on single dose NVP either in efficacy for PMTCT or reduction of NVP resistance in the mother, or both. However they appear suboptimal in that they select for NNRTI-resistant strains and therefore increase the mothers' risk of virologic failure for subsequent NNRTI-based therapy. For example, in the MASHI study [7] a total of 218 women started post partum NVP-based therapy after they had received zidovudine from 34 weeks of gestation through delivery. Of these, 112 had received single dose NVP, whilst the rest had received a placebo during labour. After 6 months of post partum treatment with a NVP-based regimen, women without prior NVP exposure were less likely to have virologic failure compared to women who had received intrapartum NVP. Strikingly, of women who started NVP-based therapy within 6 months, 41.7% from the single dose NVP group, but none from the control group, had virologic failure.

In-vivo mathematical models have been useful in exploring the evolution of drug resistance, suggesting that significant evolution can occur during treatment or before initiation of treatment [9-15]. Based on the models, the authors argued that chances of resistance evolving during treatment are small compared to chances of resistance evolving before suppressive therapy. However these studies did not explore, in any dynamically consistent framework, the emergence/accumulation of multiple mutations in a possibly non constant environment. In this study, we extend these standard models to explicitly investigate the consequences of population dynamical effects amongst common resistant mutants. We show how the deterministic dynamics of the common mutants affects the time taken to produce the rarer mutants.

We start from an ordinary differential equation (ODE)-type model of in-vivo viral replication in the deterministic regime, applicable to cell populations that are large enough for statistical fluctuations to be relatively small (wild-type and common mutant strains). We explicitly add expressions for Poisson rates for the occurrence of rare mutations. Using standard survival analysis, we compute, as a function of time, the probability of avoiding a mutation event. Furthermore, we introduce an additional timescale to the 'survival function' to capture the time over which cells infected by an unfit genome persist before being ecologically overwhelmed. This 'survival function' is a continuous state variable that is incorporated into the system of ODEs without much complexity.

We apply our modelling framework to clinically inspired scenarios. Firstly, we explore the quasi steady state that corresponds to chronic treatment in the presence of two viral populations. We characterize treatment regimes in which rates of appearance of rare mutants are either increased or decreased. Secondly, inspired by regimens used for PMTCT in resource limited countries, we investigate the transient behaviour of the model under a short perturbation of the fitness parameters, such as occurs during a short course of suboptimal therapy. Transient therapy significantly increase the hazards of rare mutations. Thirdly, we explore the interaction between a monotherapy short course and subsequent ongoing antiretroviral therapy (ART). The appendix deals with details of mutation combinatorics.

## Methods

We develop a hybrid deterministic-stochastic model of healthy and infected T cell populations. Our analysis starts with a standard multi-strain model of in-vivo viral replication that distinguishes cells infected with one of *N*_s _viral strains. These kinds of models have been used to try to understand viral evolution in the context of immune response and antiretroviral therapy [9,10,12,16,17]. For our purposes, we add a new self-consistent stochastic element to the standard deterministic model of viral evolution.

Uninfected *T *cells are produced at rate *S*_T _and die at rate *μ*_T_. Virus-producing cells, infected with strain *i*, are counted under *P*_*i *_and have a mean lifetime of 1/*μ*_P_. Mass-action (perfect mixing) contact between infected and healthy cells produces new infected cells, with a rate constant *k*_*i*_. The probability of error free transcription is given by *f *and *ε*_*ij *_is the probability of a particular mutation, that is strain *i *arising out of strain *j *from a reverse transcription error. This leads to the base model equations:

(1) $$\begin{array}{ccc} \frac{dT(t)}{dt} & = & S_{\text{T}}-T(t)\sum_{i=1}^{N_{\text{d}}}k_{i}P_{i}(t)-\mu_{\text{T}}T(t)\\ \frac{dP_{i}(t)}{dt} & = & \begin{array}{cc} fk_{i}P_{i}(t)T(t)+\sum_{\begin{array}{c} j=1\\ j\neq i \end{array}}^{N_{\text{d}}}\epsilon_{ij}k_{j}P_{j}(t)T(t)-\mu_{\text{P}}P_{i}(t), & i=1,\cdots ,N_{\text{d}} \end{array} \end{array}$$

where *N*_d _is the number of strains which are modelled by a deterministic process, i.e. those strains which are assumed to be present with sufficiently large populations for deterministic models to be sensible. We address the incorporation of rare strains shortly.

Physiologically, HIV transmission occurs either by cell-free viral particles released by infected cells, or by direct cell-to-cell contact. It has been demonstrated that cellular contacts drastically enhance productive viral transfer compared to what is observed with free virus infection [18]. Our model, like previously published models of in vivo HIV dynamics, does not have free virions. Even if free virions are physiologically important, including them for the present purposes would not change any of our conclusions as the dynamical effects appear only at very short time scales. In our basic model, *k*_*i *_is a composite fitness parameter that captures the effective cell-to-cell transmission efficiency via all mechanisms. Antiretroviral therapy with currently known drugs does not affect virion or infected-cell survival, but interferes with some stage of the viral replication cycle, i.e. reduces the values of the fitness parameter *k*_*i*_. This basic model also does not explicitly incorporate the dynamics of immune system response such as clonal expansion of effector cells or feedback linking viral and infected-cell clearance rates to the healthy cell population.

Our base parameter values are given in Table 1. We used previously estimated values for *μ*_T _= 0.02 [19] and *μ*_P _= 0.5 [20]. Since it is not possible to measure all of these directly in-vivo, some of these values are hypothetical, but they give rise to reasonable dynamics. We assume a universal, single-point-mutation rate, where the substitution rate of any base is of the order 10^-4 ^[21]. The derivation of any particular *ε*_*ij *_follows directly from combinatorial arguments outlined in the Appendix.

**Table 1:** Model parameters

Symbol	Description	Value	Source
*μ*_T_	natural healthy cell death rate	0.02 day^-1^	[40–50 days] [19]
*μ*_P_	productively infected cell death rate	0.5 day^-1^	[1–2 days] [20]
*S*_T_	supply rate of target cells	2 × 10^8 ^cells day^-1^	Estimated
*k*_*i*_	viral strain infectivity	varies	Estimated
*ε*_21_	single-point mutation rate	2.5 × 10^-5^	see Appendix
*f*	probability of error free transcription	0.37	Implied by *ε*_21_

Latently infected cells may be responsible for ongoing viral production in treated individuals, and their presence will introduce a longer timescale into a model. To capture effects of long-lived cells, we can consider the following model:

(2) $$\begin{array}{ccc} \frac{dT(t)}{dt} & = & S_{\text{T}}-T(t)\sum_{i=1}^{N_{\text{d}}}k_{i}P_{i}(t)-\mu_{\text{T}}T(t)\\ \frac{dP_{i}(t)}{dt} & = & \begin{array}{cc} fFk_{i}P_{i}(t)T(t)+\sum_{\begin{array}{c} j=1\\ j\neq i \end{array}}^{N_{\text{d}}}\epsilon_{ij}k_{j}P_{j}(t)T(t)+aL_{i}(t)-\mu_{\text{P}}P_{i}(t), & i=1,\cdots ,N_{\text{d}} \end{array}\\ \frac{dL_{i}(t)}{dt} & = & \begin{array}{cc} f(1-F)k_{i}P_{i}(t)T(t)-aL_{i}(t), & i=1,\cdots ,N_{\text{d}}. \end{array} \end{array}$$

A fraction *F *of infected cells become virus producing. The others become latent and, on average, take time 1/a to reactivate to become virus-producing cells. For simplicity, we assume that latently infected cells have a much longer lifetime than their activation time. Note that for *F *= 1, we obtain system (1). By adjusting *F *(it cannot be very realistically estimated directly from data) we can vary the importance assigned to the presence of latently infected cells, without changing the equilibrium values of healthy and virus-producing infected cells. We do not attempt to capture fine physiological details of latently infected cell dynamics, but rather the concept that these cells can be the source of new productively infected cells and hence give rise slower dynamics than a model with just virus producing infected cells.

Our main goal is to model rare mutation events which are characterized by waiting times rather than continuous processes. We consider scenarios in which the initial populations of these rare mutations are zero, and we would like to model the waiting time to their appearance. In this regime, the appearance of rare mutant strains *i *(*N*_d _<*i *≤ *N*_s_) should be modelled as a nonhomogenous Poisson process with intensity

(3) $$\begin{array}{ccc} \lambda_{i}(t)=T(t)\sum_{j=1}^{N_{d}}\epsilon_{ij}k_{j}P_{j}(t), & \text{for} & i=N_{\text{d}}+1,\cdots ,N_{\text{S}}, \end{array}$$

which captures mutations from all the deterministically modelled strains. (Recall that *N*_s _is the total number of strains.) According to standard survival analysis, the probability of there being no rare mutant of type *i*, at time *t*, given that there was none at time 0, is

(4) $$\Lambda_{i}(t)=\exp\left(-\int_{0}^{t}\lambda_{i}(\tau )d\tau \right).$$

The phylogenetic relationships amongst all strains, and the initial conditions, determine the number of continuously and stochastically modelled strains. We adopt the following computational procedure:

1. Initially run the deterministic model with populations for *N*_d _strains, and survival functions for avoiding the *N*_s _- *N*_d _rare mutants.

2. Draw a uniformly distributed random variable *R*_*i *_∈ [0, 1] for each possible rare mutation event.

3. When Λ_*i *_reaches *R*_*i *_the appearance of mutant *i *occurs.

4. If the mutant appears into an environment in which it is fit enough to thrive, pause the simulation.

5. Add one cell of the new rare mutant.

6. Resume running the new deterministic model with ${N^{\prime }}_{\text{d}}$ = *N*_d _+ 1 strains.

This piecewise deterministic model is hardly more complex than a purely deterministic model. The abrupt changes to the evolving process transforms the ODE system into differential equations involving impulse effects (impulsive differential equations) [22]. The difference between our computational approach and previously considered schemes of which we are aware, is that in our scheme, mutation hazards are derived from explicit deterministic model state variables, and also, they depend on mutational pathways, whereas, for example, in Nowak *et al *[23], the probability of generating a new mutant is proportional to the total virus population.

Note that in the computational procedure just outlined, we have only explicitly modelled the consequences of those rare genomes which have a fitness above a critical value. Of course, the appearance of low fitness mutations is also possible. We now propose that genomes with sub-critical fitness, which do not give rise to explicitly modelled populations, survive, presumably in trace quantities, for a typical time (which we call Δ) before they are driven to extinction. If there is an environmental shift during this persistence period, such as initiation of therapy that strongly suppresses the other genomes, this one can then begin to thrive and grow in the same manner as any mutation which arises into an initially favourable environment. If the perfect-mixing model is assumed to be valid on all size scales, this new timescale would simply be the lifetime of the infected cell bearing the new genome, as an unfit variant will be unlikely, under a fully stochastic treatment, to produce daughter cells. However, it is far from certain that this simple view captures the dynamics surrounding rare mutations. A small local cluster of cells bearing the new genome may have a good chance of arising from the seminal mutation, but then be almost certain to be overwhelmed ecologically within a typical time as mixing or directly competing with fitter variants occurs. Since we do not know what this time may be, we simply note the crucial role it plays in our modified survival analysis. Now, instead of simply considering the probability that the mutant has never occurred since time 0, as in equation (4), we consider the probability that a rare mutant has not occurred in the most recent time interval of size Δ, i.e.

(5) $$\Lambda_{\Delta }(t)=\exp\left(-\int_{t-\Delta }^{t}\lambda (\tau )d\tau \right).$$

This new state variable is just the probability that the relevant mutant genome is absent at time *t*. The smallest physically feasible value of Δ is the lifetime of an infected cell (as noted above for the case where the rare mutant produces no daughter cells from the seminal mutation) and the largest feasible value is greater than the expected survival time of the infected individual (if the genome is significantly banked into a latently infected cell population) i.e. essentially infinite for practical purposes. It is particularly relevant when we model environmental shifts, such as the start or end of an antiretroviral (ARV) regimen. We will demonstrate scenarios in which the presence or absence of a 'currently unfit' genome, at the moment of initiation of therapy, can have an impact on rates of treatment failure.

## Results and discussion

We now apply, to clinically inspired scenarios, the survival analysis of the model presented in the previous section. The particular model implementations are in certain respects simplistic preliminary work, but they demonstrate the kinds of questions that can be seriously investigated within this framework. We use a model with two continuously variable strains (wild-type and common mutant) and a waiting time for the appearance of the third strain (rare mutant). In the absence of treatment, the wild-type strain is dominant and the mutant subpopulation is present in trace quantities, of the order of the mutation rate.

We are interested in modelling mutations that occur rarely i.e. those that do not typically exist at most points in time. Consider a mutant which differs from the wild-type by three single-point mutations (say M1, M2 and M3) and from a common mutant (M1) by two-point mutations (M2 and M3). We assume that strains bearing just M2 or M3, or any two of M1, M2 and M3 are highly fitness compromised i.e. the M1 M2 M3 (*P*_3_) are all compensatory mutations. We use this particular phylogeny to illustrate the application of our method. Adding a rare mutant to a two-strain deterministic model as strain *i *= 3 gives the Poisson rate

(6) *λ*_3_(*t*) = *T*(*t*)(*ε*_31_*k*_1_*P*_1_(*t*) + *ε*_32_*k*_2_*P*_2_(*t*)),

which shows how a rare mutant variant can arise through a number of pathways, such as sequential single-point mutations or simultaneous higher-order mutations.

First, we consider a quasi steady state scenario corresponding to chronic treatment, then we model a short course of monotherapy, followed, after some delay, by initiation of chronic therapy. Important interactions between the two regimens are captured by the newly introduced state variable Λ_Δ_.

### Chronic treatment/Steady state

We start by analyzing the steady-state dynamics of the two continuously modelled strains in the absence of a rare mutant. The choice of parameter *F*, which introduces latently infected cells, does not affect this analysis. As is typical with these simple in-vivo models, our two-strain deterministic model has two steady states: the uninfected steady state and a unique infected steady state which is either physical (a positive number of infected cells) or unphysical (a negative number of infected cells) depending on the fitness parameters. For a two-strain deterministic model (with wild-type strain *P*_1 _as the initially infecting strain and common mutant strain *P*_2_, a result of single-point mutations from the wild-type strain), the exact equilibrium solution is given by

(7) $$\begin{array}{lll} \bar{T} & = & \frac{\mu_{\text{P}}\left(f(k_{1}+k_{2})-\sqrt{f^{2}{\left(k_{1}+k_{2}\right)}^{2}-4(f^{2}-\epsilon_{21}^{2})k_{1}k_{2}}\right)}{2\left(f^{2}-\epsilon_{21}^{2}\right)k_{1}k_{2}}\\ {\bar{P}}_{1} & = & \frac{\left(S_{\text{T}}-\mu_{\text{T}}\bar{T}\right)\left(\mu_{\text{P}}-fk_{2}\bar{T}\right)}{k_{1}\bar{T}\left(\mu_{\text{P}}-(f-\epsilon_{21})k_{2}\bar{T}\right)}\\ {\bar{P}}_{2} & = & \frac{\epsilon_{21}k_{1}{\bar{P}}_{1}\bar{T}}{\mu_{\text{P}}-fk_{2}\bar{T}}, \end{array}$$

where *k*_1 _> *k*_2_. An approximate, much simpler, equilibrium solution can be derived directly from the exact equations (7) by setting $\epsilon_{21}^{2}$ and other higher-order terms to zero.

(8) $$\begin{array}{lll} \bar{T} & = & \frac{\mu_{\text{P}}}{fk_{1}}+O(\epsilon_{21}^{2})\\ {\bar{P}}_{1} & = & \frac{\mu_{\text{T}}}{k_{1}}({R^{\prime }}_{01}-1)\left(1-\frac{\epsilon_{21}k_{2}}{f(k_{1}-k_{2})}\right)+O(\epsilon_{21}^{2})\\ {\bar{P}}_{2} & = & \frac{\epsilon_{21}\mu_{\text{T}}}{f(k_{1}-k_{2})}({R^{\prime }}_{01}-1)+O(\epsilon_{21}^{2}), \end{array}$$

where the wild-type is the fitter strain (*k*_1 _> *k*_2_). The basic reproductive ratio of strain *i *is given by

(9) $$\begin{array}{cc} {R^{\prime }}_{0i}=\frac{fS_{\text{T}}k_{i}}{\mu_{\text{P}}\mu_{\text{T}}} & i=1,2. \end{array}$$

We are interested in modelling the regime where ${R^{\prime }}_{0i}$ is always greater than one in the absence of therapy, since our main focus is on persistent infection. Note that

(10) $${\bar{P}}_{2}=\frac{\epsilon_{21}k_{1}}{f(k_{1}-k_{2})}{\bar{P}}_{1}+O(\epsilon_{21}^{2}).$$

The less-fit strain is present in trace quantities that will be very difficult to detect by standard clinical assays, even if the fitness difference is marginal. This is a modified version of the usual ecological phenomenon that two species in a single niche do not coexist even with very similar fitness; one dominates and drives the other to extinction. The non-extinction of the less-fit quasispecies observed in this case results from the high mutation rate, which leads to waiting times between mutation events that are very small compared to the lifetimes of productively infected cells, so that the subdominant species persists in significant quantities. Given realistic orders of magnitude for infected cell populations (10^9^) and lifetimes (a day), and the mutation rates between strains that differ by a single base mutation (10^-5^), certain minority populations (single-point and double-point mutations relative to a dominant wild type) are large enough to be modelled deterministically.

The mean waiting time to the occurrence of a rare mutation according to the Poisson rate (equation (6)) before treatment (evaluated at the pre-treatment equilibrium state) is given by

(11) $$\langle \tau_{w}\rangle ={\left[\bar{T}\left(\epsilon_{31}k_{1}{\bar{P}}_{1}+\epsilon_{32}k_{2}{\bar{P}}_{2}\right)\right]}^{-1}\approx {\left[\frac{S_{\text{T}}({R^{\prime }}_{01}-1)}{{R^{\prime }}_{01}}\left(\epsilon_{31}+\frac{(\epsilon_{32}-\epsilon_{31})\epsilon_{21}k_{2}}{f(k_{1}-k_{2})}\right)\right]}^{-1}.$$

**Figure 1:**
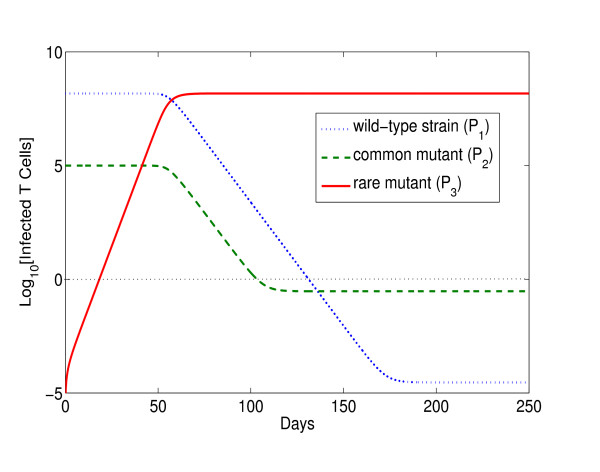
**Growth of a rare mutant in the deterministic model**. Growth of a rare mutant strain (ie. with no waiting time) in the deterministic model. The initial value of the rare mutant (*P*_3_) is zero whereas viral strains: wild-type (*P*_1_) and common mutant (*P*_2_) begin their dynamics from the steady state. The rare mutant is immediately produced continuously and grows according to its fitness advantage. The time required to attain one cell infected by this rare mutant is of the order of weeks. In these simulations, the differential fitness parameters are given by (*k*_3_, *k*_2_, *k*_1_) = (2*k*_1_, 0.9*k*_1_, *k*_1_) where *k*_1 _= 2 × 10^-8^; that is, the environment favours the rare mutant to outgrow existing viral variants.

It is important to understand how fundamentally different this result (and reality) is from a what can be obtained in a model which treats all strains deterministically. Mathematically, it is perfectly sensible to define a model with any number of deterministically strains, as per the basic model above, and to try to capture the 'rare' mutants by using suitably small mutation rates. When a purely deterministic model runs from an initial condition in which the fitter strain is absent, this absent strain is immediately produced continuously. The new strain then grows according to its fitness advantage (see figure 1). Thus, the time taken for it to reach some proportion of the total infected cell population is deterministic, and substantially dominated by the dynamical interaction of the two strains. The time required to attain one cell infected by the new strain (*P*_3 _= 1) can be derived by solving *P*_3_(*t*) = 1 from an initial value of *P*_3 _= 0, and using the dynamical equation

(12) $$\begin{array}{lll} \frac{dP_{3}(t)}{dt} & = & fk_{3}P_{3}(t)T(t)+T(t)\left(\epsilon_{31}k_{1}P_{1}(t)+\epsilon_{32}k_{2}P_{2}(t)\right)-\mu_{\text{P}}P_{3}(t)\\ & = & {\tilde{\lambda }}_{3}+\mu_{\text{P}}(\gamma -1)P_{3}(t) \end{array}$$

where

(13) $$\begin{array}{ccc} {\tilde{\lambda }}_{3}=\frac{S_{\text{T}}({R^{\prime }}_{01}-1)}{{R^{\prime }}_{01}}\left(\epsilon_{31}+\frac{(\epsilon_{32}-\epsilon_{31})\epsilon_{21}k_{2}}{f(k_{1}-k_{2})}\right) & \text{and} & \gamma =\frac{k_{3}}{k_{1}}>1. \end{array}$$

This implements the assumption that the other cell populations are not significantly perturbed from their initial values over the time it takes to produce one cell of the rare mutant. Then

(14) $$\langle \tau_{1}\rangle =\frac{1}{\mu_{\text{P}}(\gamma -1)}\ln\left(1+\frac{\mu_{\text{P}}(\gamma -1)}{{\tilde{\lambda }}_{3}}\right).$$

For the chosen parameter values (Table 1), the time required to attain one cell infected by the new strain is of the order of weeks (see figure 1). On the other hand, the explicitly modelled mean waiting time (⟨*τ*_*w*_⟩) to the occurrence of the new mutation according to the constant Poisson rate ${\tilde{\lambda }}_{3}$ is of the order of years. It seems to us that the latter is a reasonable model of rare events and the former is fundamentally flawed.

We now return to the stochastic waiting time model, which at the pre-treatment equilibrium state, has a waiting time to the occurrence of a rare mutant of the order of years for the chosen parameter values given in Table 1. We are interested in the impact of long term treatment on these waiting times. Let treatment efficacy on strain *i *be denoted by *ξ*_*i *_∈ [0, 1], so that the infectivity parameter during treatment is ${k^{\prime }}_{i}$ = *ξ*_*i*_*k*_*i*_.

**Figure 2:**
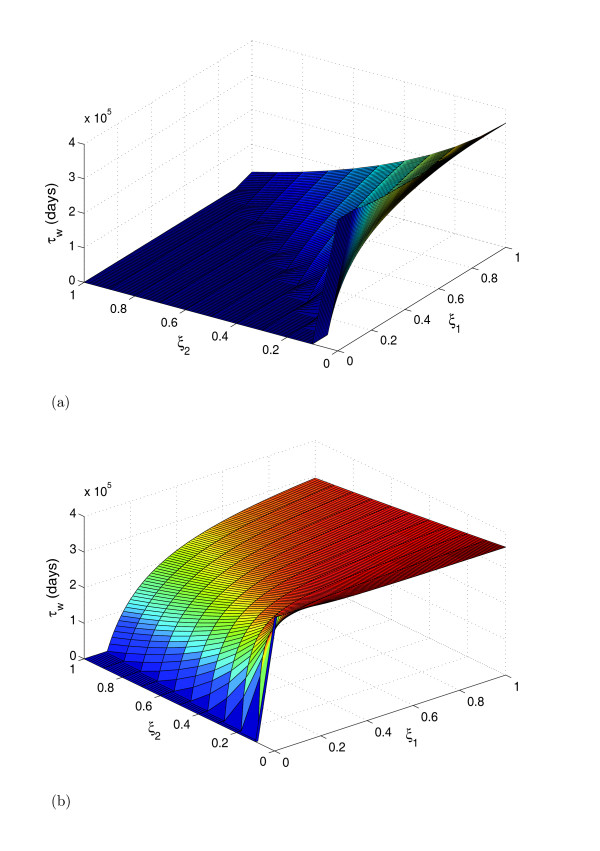
**Surface plots of waiting times as a function of drug efficacy**. Surface plot showing the waiting times to the occurrence of rare mutations as a function of drug efficacy on viral strains (*ξ*_*i*_) for (a) *k*_2 _= 0.9*k*_1 _and (b) *k*_2 _= 0.1*k*_1 _where *k*_1 _= 2 × 10^-8^. The point ((*ξ*_1_, *ξ*_2_) = (0, 0)) represents potent treatment that results in viral elimination. Less effective selection pressure or treatment that successfully suppresses both viral subpopulations results in increased waiting times or even guarantees the non-occurrence of rare mutations. Suboptimal treatment that suppresses the wild-type strain but barely affects the common mutant leads to dramatic reduction in waiting times to the occurrence of rare mutations. On the other hand, treatment that affects the common mutant but allows continuation of wild-type strain replication, increases the waiting time to the occurrence of a rare mutant.

In figure 2, we show the surface plots of the waiting times to the occurrence of rare events as a function of drug efficacy on viral strains. A key result is that waiting times are significantly smaller when the common mutant is only marginally less fit, than when there is a large fitness cost. Note that these plots describe a relationship between 'clinical' parameters (waiting times) and pharmacological parameters (drug efficacies) which are difficult to determine in-vivo. On the other hand, quantitation of plasma HIV RNA can be performed to determine viral populations which in turn can be used as alternative parameters to calculate the waiting times to the occurrence of rare mutations. We demonstrate this by introducing parameters which express treatment effectiveness at the level of changes in the equilibrium viral loads. Let the treated equilibrium values of the wild-type and the mutant-strain-infected cell populations, relative to the pre-treatment wild-type infected cell level, be given by

(15) $$\begin{array}{ccc} F_{w}=\frac{{\bar{P}}_{1}({k^{\prime }}_{1},{k^{\prime }}_{2})}{{\bar{P}}_{1}(k_{1},k_{2})} & \text{and} & F_{m}=\frac{{\bar{P}}_{2}({k^{\prime }}_{1},{k^{\prime }}_{2})}{{\bar{P}}_{1}(k_{1},k_{2})}, \end{array}$$

respectively. Recalling that in the untreated state, the viral load is strongly dominated by the wild type (${\bar{P}}_{1}$(*k*_1_, *k*_2_)), this notation facilitates comparisons between the treated and untreated states, both in terms of overall viral suppression, and selection between strains.

Disruption of the pre-treatment equilibrium state (*F*_*w *_= 1 and *F*_*m *_≪ 1) by therapy leads to different possible effects on the "benchmark" (pre-treatment) waiting time. The limiting case scenarios of interest are

1. Therapy suppresses the mutant subpopulation (*F*_*m *_→ 0) but allows the dominant wild-type strain to replicate relatively unhindered (*F*_*w *_≈ 1); this increases the waiting times to the occurrence of a new strain. In other words, even though the total viral load is barely affected, there is a benefit in terms of impaired viral evolution.

2. Treatment is optimal against the wild-type strain (*F*_*w *_→ 0) but barely affects the common mutant i.e.

(16) $$F_{m}\to \frac{k_{1}}{k_{2}}\left(\frac{{R^{\prime }}_{02}-1}{{R^{\prime }}_{01}-1}\right),$$

where ${R^{\prime }}_{0i}$ is strain *i *reproductive ratio. This leads to a dramatic reduction in waiting times to the appearance of rare mutations, i.e. the much more rapid emergence of the rare mutant.

3. Treatment is optimal; that is, treatment that successfully suppresses both viral subpopulations ((*F*_*w*_, *F*_*m*_) → (0, 0)). This essentially guarantees the non-occurrence of rare mutations.

### Transient Nevirapine monotherapy

**Figure 3:**
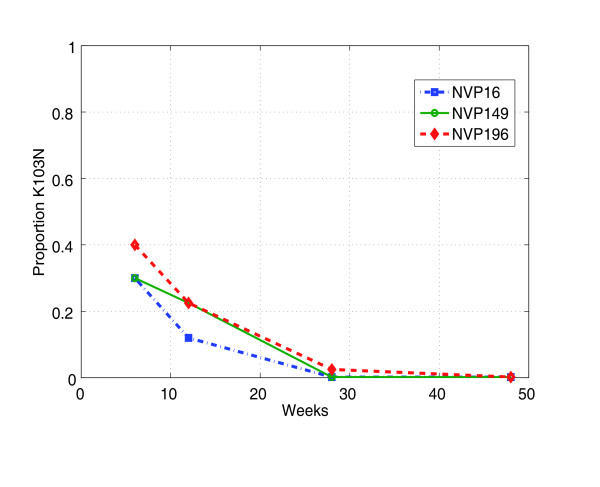
**Clinical nevirapine data**. Relative fractions of K103N variants in maternal plasma viral RNA after single dose nevirapine for three individual women (NVP16, NVP19 and NVP196). We read data off a chart published in [24]. In this study, the relative abundance of K103N declined to undetectable levels by 12 months [24].

We use our piecewise deterministic model to explore the consequences of transient increases in the relative frequency of common mutations (such as K103N) on the occurrence of rarer mutations, during and after short-course monotherapy. To obtain curves resembling the K103N decay data shown in figure 3[24], we incorporate a population of latently infected (i.e. long-lived and non-virus-producing) cells [25,26] that are activated to productively infected cells on a timescale of 2 to 3 weeks, by setting *F *= 0.9.

**Figure 4:**
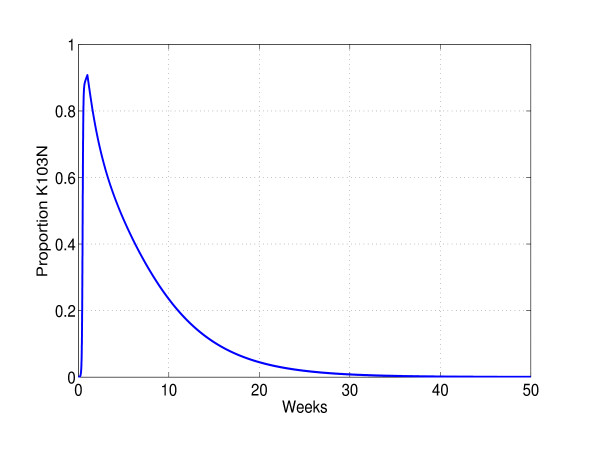
**Transient increase of common mutations**. Relative frequency of K103N during and after 7 days of idealized treatment. Short-course highly selective therapy results in dramatic increases in pre-existing resistant viral variants. Withdrawal of therapy results in a slow decline of the subpopulation. We assume that 10% of infected cells become long-lived infected cells and are activated after 2 weeks i.e. we set *F *= 0.90 and *a *= 1/14.

**Figure 5:**
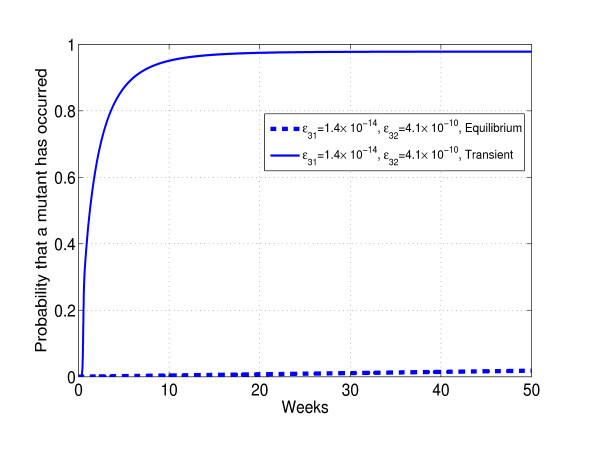
**Probabilities of observing a new mutation**. The cumulative probabilities of observing a new mutation in the absence (Equilibrium) and presence (Transient) of drug pressure. Model choices which produce a transient of a magnitude and duration shown in figure 4 lead to significant acceleration of viral evolution. In the absence of selective pressure chances of observing a new mutant are negligible.

First we explore the effect of a single short course of highly selective treatment on waiting times to the appearance of rare mutations. Initiating suboptimal therapy results in dramatic increase of the common resistant mutant (K103N) population and an equally dramatic decrease in the wild-type strain (K103) population. When pressure of therapy is discontinued, the common mutant population declines to pre-treatment levels. At every time point during and after short course therapy, we evaluate the cumulative probability of a rare mutation having occurred. Figure 4 illustrates the transient increase and decline in the proportion of a common mutant, and figure 5 shows the corresponding cumulative probabilities of observing a rare mutant. We compare the cumulative probability under transient treatment to the case in which therapy is not given at all. For the chosen parameters, the probability of observing a rare mutation, within a year in the absence of therapy, is negligible. However, transient therapy dramatically increases this probability.

**Figure 6:**
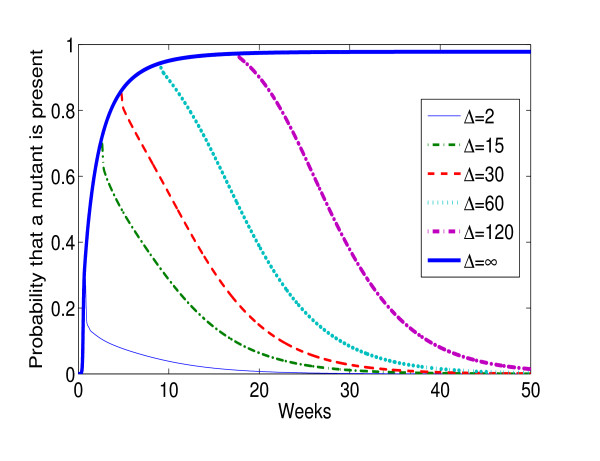
**Probability that a relevant mutant genome is present**. The probability of there existing a mutant which persists for a time Δ after a seminal mutation, plotted as a function of time from a brief period of selective pressure (such as single dose NVP). Plots are shown for a number of values Δ ranging from 2 days to 350 (taken as ∞) days. The other parameter values are as in figure 4.

Next we explore the interaction between a monotherapy short course and subsequent continuous therapy, as was investigated in the MASHI study. We evaluate the probability that a rare mutant is currently present at time *t*, given various possible values of the rare mutant persistence timescale Δ (see figure 6). For values of Δ less than 60 days, the probability of a rare mutant being present, at some point more than 6 months after the single dose of NVP, is small (< 5%) i.e. cells infected by the new genome are unlikely to be present, and hence pose a low residual risk if the mother is put on treatment more than six months after single dose Nevirapine for PMTCT. This is not inconsistent with the clinical findings from the MASHI study [7]. The reality is presumably more complex than what our model can capture, but little is known about the persistence of unfit mutants. It has been observed that resistant genomes may persists, even at undetectable level, for prolonged periods [27,28]. It makes sense that initiating therapy in the presence of a therapy defeating mutant, or an immediate precursor to such a mutant (even at levels too low for detection by typical assays), will reduce chances for treatment success.

## Conclusion

We have considered a number of more-or-less standard deterministic multi-strain models of in-vivo viral dynamics, which are tunable to produce scenarios like a chronic ARV regimen, and a short course of monotherapy. We have adjoined a stochastic component to these models, in the form of a 'sliding window' survival analysis, which substantially expands the possible analyses of rare strain dynamics within the framework of ordinary differential equations.

We have considered scenarios which capture the concepts of a dominant wild-type strain, a relatively unimpaired 1 base mutant, a number of unviable 2 base mutants, and the possibility of compensatory mutations which lead to a treatment defeating 3 base mutant that is reachable by different pathways, *which have different relative importance at various stages during transient dynamics*. Different phylogenies, together with different chosen fitness parameter values, will result in different numbers of deterministically and stochastically modelled strains and pathways, all of which can immediately be accommodated into our general model.

The transient increases in common subpopulations of cells infected by mutant genomes produced by the short course of antiviral therapy affects waiting times to the appearance of rare mutations, conceived as differing more from the wild type than from the deterministically modelled primary mutant. Over a range of model choices which produce a transient of the order (size and duration) of that known to occur under nevirapine monotherapy used for PMTCT, there is significant acceleration of viral evolution – even from just the short course alone. This effect is suspected, but not unambiguously observed, from clinical studies.

A further important set of questions arises about the risks associated with initiation of chronic therapy (HAART) a short while after the suboptimal transient regimen like for PMTCT. Our newly proposed additional timescale Δ, representing the persistence of a new genome in an infavourable environment has a substantial impact on the rates of treatment failure.

These models demonstrate that even transient subpopulations of common mutants which appear to fade are associated with accelerated appearance of rarer mutations. Further work which should be performed includes 1) variations on these models which are designed to capture precise genetic differences (and hence realistic pathways and mutation rates) between sets of quasispecies being directly observed in studies utilising highly sensitive assays, and 2) biological investigation into the dynamics of small populations of new mutants, which these models summarise into the timescale Λ_Δ_.

## Competing interests

The authors declare that they have no competing interests.

## Authors' contributions

TS and AW conceived, designed and analyzed the model, interpreted the model results and wrote the manuscript.

## Appendix: mutation combinatorics

We relate the HIV mutation process parameters *ε*_*ij *_and *f *to an underlying single-point-mutation process.

The error rate per site for HIV reverse transcriptase (for any given nucleotide A, C, G and T) is assumed to be 10^-4 ^[21], so that the rate of change to any of the three alternatives (for example substitution of A by C, G or T) is given by *η *= $\frac{1}{3}$ × 10^-4^. The probability that a site within a gene will remain unchanged after reverse transcription (i.e. A → A, C → C, T → T or G → G) is given by (1 – 3*η*). Then the probability of a particular mutation (strain *j *→ strain *i*), where *i *and *j *differ by precisely *m *point mutations, given a genome length *L *(approximately 10^4 ^bases for HIV) is given by

(17) *ε*_*ij *_= (1 – 3*η*)^*L*-*m*^*η*^*m *^≈ (1 – 3*η*)^*L*^*η*^*m *^≡ *fη*^*m*^,

where *m *<<*L *and 1 – 3*η *≈ 1. Note that *f *= (1 – 3*η*)^*L *^≈ 0.37 is the probability of error free replication. For example, we consider particular point mutations at codon 103 of reverse trancriptase gene that are associated with 103K/N viral populations. Given that AAA & AAG code for K and AAC & AAT code for N, the rate of lysine (K) substitutions by asparagine (N) at this codon is given by

(18) ℙ(K → N) = 2*fη*.

The single point mutation rate at this codon using equation (18) is given by *ε*_21 _= 2.5 × 10^-5^. Then, using equation (17), we have *ε*_31 _= *fη*^3 ^≈ 1.4 × 10^-14 ^and *ε*_32 _= *fη*^2 ^≈ 4.1 × 10^-10^.
